# *CDKN2A* germline alterations and the relevance of genotype-phenotype associations in cancer predisposition

**DOI:** 10.1186/s13053-021-00178-x

**Published:** 2021-03-25

**Authors:** Sock Hoai Chan, Jianbang Chiang, Joanne Ngeow

**Affiliations:** 1 Cancer Genetics Service, Division of Medical Oncology, National Cancer Centre Singapore, Singapore, 169610 Singapore; 2 Oncology Academic Clinical Program, Duke-NUS Medical School, Singapore, 169857 Singapore; 3 Lee Kong Chian School of Medicine, Nanyang Technological University Singapore, Singapore, 308232 Singapore

**Keywords:** *CDKN2A*, Cancer predisposition, p16^INK4A^, p14^ARF^

## Abstract

Although *CDKN2A* is well-known as a susceptibility gene for melanoma and pancreatic cancer, germline variants have also been anecdotally associated with a broader range of neoplasms including neural system tumors, head and neck squamous cell carcinomas, breast carcinomas, as well as sarcomas. The *CDKN2A* gene encodes for two distinct tumor suppressor proteins, p16^INK4A^ and p14^ARF^, however, the independent association of germline alterations affecting these two proteins with cancer is under-appreciated. Here, we reviewed *CDKN2A* germline alterations reported among individuals and families with cancer in the literature, specifically addressing the cancer phenotypes in relation to the molecular consequence on p16^INK4A^ and p14^ARF^. While melanoma is observed to associate with variants affecting both p16^INK4A^ and p14^ARF^ transcripts, it is noted that variants affecting p14^ARF^ are more frequently observed with a heterogenous range of cancers. Finally, we reflected on the implications of this inferred genotype-phenotype association in clinical practice and proposed that clinical management of *CDKN2A* germline variant carriers should involve dedicated cancer genetics services, with multidisciplinary input from various healthcare professionals.

## Background

*CDKN2A* (cyclin dependent kinase inhibitor 2A, OMIM 600160) is a tumor suppressor gene that encodes for two proteins, namely p16^INK4A^ and p14^ARF^, critical for the regulation of cell cycle pathways. Genetic and epigenetic alterations inactivating *CDKN2A* are frequently encountered in a myriad of cancers, with base sequence-altering events more common in cancer types such as melanoma, head and neck squamous cell carcinoma (HNSCC), pancreatic cancer, lung cancer, esophageal cancer, and glioblastoma multiforme (GBM) [1–3]. Germline alterations in *CDKN2A* are most frequently associated with predisposition to melanoma and pancreatic cancer [4–8], detected through gene-panel testing in about 38% of melanoma-prone families [6, 9] but there have been sporadic reports implicating susceptibility to other neoplasms such as neural system tumors (NSTs), breast cancer, multiple myeloma, HNSCC, and sarcoma [10–18]. It is plausible that the varying cancer types reported with *CDKN2A* genetic alterations can be distinguished by the different variant effects on p16^INK4A^ and p14^ARF^, although evidence to date are limited and conflicting [12, 13, 16, 19–21]. Here, we reviewed the spectrum of *CDKN2A* germline variants and associated neoplasms reported in literature, focusing on the relationship between distinct variant consequences on p16^INK4A^/p14^ARF^ with the reported phenotypes. Variants evaluated include those detected in affected individuals through sequencing and/or classified as pathogenic or likely pathogenic in ClinVar database (version 2020-09-08, https://www.ncbi.nlm.nih.gov/clinvar/) without conflicts in interpretation.

### p16^INK4A^/p14^ARF^ locus in the CDKN2A gene

The *CDKN2A* gene spans 27.5 kb on chromosome 9p21 and is associated with over 10 transcript variants, of which the largest two encode for p16^INK4A^ and p14^ARF^ [22]. p16^INK4A^ is a 156 amino acid protein translated from a transcript of three exons (exons 1α,2,3; RefSeq NM_000077), known to negatively regulate cell cycle progression through inhibition of cyclin-dependent kinases [23]. The largest transcript produces p14^ARF^ (RefSeq NM_058195), a 132 amino acid protein, encoded via an alternative open-reading frame and first exon (exon 1β), with an established role of promoting p53 function through sequestration of MDM2 [24]. Consequently, p16^INK4A^ and p14^ARF^ are distinct proteins with different roles and no sequence homology, sharing only the use of same exons 2 and 3. Notably, although both tumor suppressors are encoded by three exons of similar size (exon1α: 421 bp, exon 1β: 486 bp, exon 2: 307 bp, exon 3: 490 bp), the bulk of translated sequence is localized to exon 1α/1β and exon 2.

### Spectrum of p16^INK4A^/p14^ARF^ variants associated with neoplasms

There are differences in molecular consequences of *CDKN2A* variants reported in literature on p16^INK4A^ and p14^ARF^, which is expected given the use of different open-reading frames. Most of the p16^INK4A^-affecting variants are missense changes (28/55) followed by protein-disrupting variants (20/55, including truncating and null effects), occurring on exon1α and exon 2 (Table 1). In comparison, almost one-third of these reported variants fall within intron 1 of p14^ARF^ transcript corresponding to exon1α of p16^INK4A^, followed by missense (16/62) and protein-disrupting (13/62) changes, which are mostly concentrated in exon 2 of p14^ARF^. Due to the difference in transcript architecture, there is an overall higher likelihood of encountering variants outside of protein sequence-coding regions (e.g. intronic, 3-prime untranslated region) in p14^ARF^ compared to p16^INK4A^.

**Table 1:** Pathogenic/likely pathogenic germline variants in *CDKN2A* affecting p16^INK4A^, p14^ARF^ transcripts and the associated neoplasms reported in the literature

Effect on p14ARF (Transcript: NM_058195)				Effect on p16INK4A (Transcript: NM_000077)				Neoplasms reported in variant carriers									References
Exon/ intron	Nucleotide change	Protein change	Variant effect	Exon/ intron	Nucleotide change	Protein change	Variant effect	MEL	PCa	NST	SARC	BrCa	HNSCC	LCa	NFB	Other neoplasms	
Ex 1β	Del of Exon 1	–	null	–	–	–	–	/								uterine adenocarcinoma, thyroid adenomata, chest wall neurilemmoma, pituitary macroadenoma	[21, 25]
Ex 1β	c.47G > A	Gly16Asp	MS	–	–	–	–	/									[20]
Ex 1β	c.60_61insCGGCCCGCCGCGAGTG	Val22Profs*46	PT	–	–	–	–	/									[19]
Ex 1β	c.97dup	Glu33Glyfs*30	PT	–	–	–	–									Bladder ca.	[26]
Ex 1β	c.161G > A	Arg54His	MS	–	–	–	–	/									[27]
Ex 1β	c.193G > C	Gly65Arg	MS	–	–	–	–	/				/					[25, 28, 29]
In 1	c.193 + 1G > A	n.d.	Splice^a^	–	–	–	–	/									[25, 27, 29–31]
In 1	c.194-3653G > T	n.d.	Int	Ex 1α	c.-34G > T	–	5’UTR	/									[25, 29, 32–35]
In 1	c.194-3635dup	n.d.	Int	Ex 1α	c.-16_8dup	Ala4_Pro11dup ^b^	In-frame INS	/								Multiple myeloma, brain tumor, colorectal ca.	[14, 36–40]
In 1	c.194-3585C > A	n.d.	Int	Ex 1α	c.35C > A	Ser12*	PT	/									[41, 42]
In 1	c.194-3576G > A	n.d.	Int	Ex 1α	c.44G > A	Trp15*	PT	/									[25, 35, 42–44]
In 1	c.194-3573 T > G	n.d.	Int	Ex 1α	c.47 T > G	Leu16Arg	MS	/	/								[4, 25, 29, 45, 46]
In 1	c.194-3573 T > C	n.d.	Int	Ex 1α	c.47 T > C	Leu16Pro	MS	/	/								[9, 45–48]
In 1	c.194-3553G > A	n.d.	Int	Ex 1α	c.67G > A	Gly23Ser	MS	/									[45]
In 1	c.194-3552G > A	n.d.	Int	Ex 1α	c.68G > A	Gly23Asp	MS	/									[25, 48–51]
In 1	c.194-3549G > C	n.d.	Int	Ex 1α	c.71G > C	Arg24Pro	MS	/	/		/						[25, 32, 34, 51–53]
In 1	c.194-3541G > T	n.d.	Int	Ex 1α	c.79G > T	Glu27*	PT	/								Neuroblastoma	[25, 54, 55]
In 1	c.194-3525 T > C	n.d.	Int	Ex 1α	c.95 T > C	Leu32Pro	MS	/	/		/						[18, 25]
In 1	c.194-3514delG	n.d.	Int	Ex 1α	c.106delG	Ala36Argfs*17	PT	/					/				[15]
In 1	c.194-3489_194-3488insAA	n.d.	Int	Ex 1α	c.131_132insAA	Tyr44*	PT	/									[56–58]
In 1	c.194-3488C > G	n.d.	Int	Ex 1α	c.132C > G	Tyr44*	PT	/									[56–58]
In 1	c.194-3488del	n.d.	Int	Ex 1α	c.132del	Tyr44*	PT	/	/								[59]
In 1	c.194-3478C > A	n.d.	Int	Ex 1α	c.142C > A	Pro48Thr	MS	/									[25]
In 1	c.194-3477C > T	n.d.	Int	Ex 1α	c.143C > T	Pro48Leu	MS	/									[60]
In 1	c.194-3474 T > G	n.d.	Int	Ex 1α	c.146 T > G	Ile49Ser	MS	/	/		/						[18, 25]
In 1	c.194-3472C > T	n.d.	Int	Ex 1α	c.148C > T	Gln50* ^c^	PT	/	/								[32]
In 1	c.194-3471A > C	n.d.	Int	Ex 1α	c.149A > C	Gln50Pro	MS	/									[52]
In 1	c.194-69C > T	n.d.	Int	In 1	c.151-69C > T	–	Int	/ (Uvl)									[61]
In 1	c.194-2A > G	n.d.	Splice	In 1	c.151-2A > G	n.d.	Splice				/						[18]
In 1	c.194-1G > C	n.d.	Splice^a^	In 1	c.151-1G > C	n.d.	Splice^a^	/		/		/			/	Osteochondroma	[11, 13, 62]
Ex 2	c.202G > C	Asp68His	MS	Ex 2	c.159G > C	Met53Ile	MS	/									[32, 34]
Ex 2	c.202G > A	Asp68Asn	MS	Ex 2	c.159G > A	Met53Ile	MS	/	/								[32]
Ex 2	c.210G > T	Gln70His	MS	Ex 2	c.167G > T	Ser56Ile	MS	/									[34, 35, 48, 63–65]
Ex 2	c.215C > T	Pro72Leu	MS	Ex 2	c.172C > T	Arg58* ^d^	PT	/									[25]
Ex 2	c.219 T > G	Ser73Arg	MS	Ex 2	c.176 T > G	Val59Gly	MS	/									[4, 30, 33, 48, 64, 66–68]
Ex 2	c.237 T > C	Ala79=	Silent	Ex 2	c.194 T > C	Leu65Pro	MS				/						[18, 25]
Ex 2	c.245_246delinsTT	Arg82Leu	MS	Ex 2	c.202_203delinsTT	Ala68Leu	MS	/	/								[25]
Ex 2	c.255A > G	Gln85=	Silent	Ex 2	c.212A > G	Asn71Ser	MS	/									[4, 59, 69, 70]
Ex 2	c.256C > A	Leu86Met	MS	Ex 2	c.213C > A	Asn71Lys	MS	/				/		/		Multiple myeloma	[17, 25]
Ex 2	c.262C > T	Arg88*	PT	Ex 2	c.219C > T	Ala73=	Silent									Stomach ca.	[26]
Ex 2	c.268_286del / c.269_287del	Arg90Valfs*76	PT	Ex 2	c.225_243del / c.226_244del	Ala76Cysfs*64 ^e^	PT	/	/				/			Uterine carcinomasarcoma	[18, 25, 32, 44, 71–76]
Ex 2	c.283_296del	Thr95Leufs*61	PT	Ex 2	c.240_253del	Pro81Cysfs*34	PT	/	/						/	Papillary thyroid ca., uterine tumors	[77]
Ex 2	c.302C > T	Pro101Leu	MS	Ex 2	c.259C > T	Arg87Trp	MS	/									[33, 55, 63, 78, 79]
Ex 2	c.303G > C	Pro101=	Silent	Ex 2	c.260G > C	Arg87Pro	MS	/					/	/			[10, 25, 69, 80]
Ex 2	c.305G > T	Gly102Val	MS	Ex 2	c.262G > T	Glu88*	PT	/									[33, 81]
Ex 2	c.326del	Gly109Valfs*63	PT	Ex 2	c.283del	Val95Trpfs*51	PT		/								[46]
Ex 2	c.451_454dupGGTG	Ala152Glyfs*51	PT	Ex 2	c.285_288dupGGTG	Leu97Glyfs*24	PT	/								Low grade neuroepithelial tumor	[53]
Ex 2	c.339G > C	Pro113=	Silent	Ex 2	c.296G > C	Arg99Pro	MS	/	/								[25]
Ex 2	c.344G > T	Arg115Leu	MS	Ex 2	c.301G > T	Gly101Trp	MS	/			/						[18, 25, 32, 33, 67]
Ex 2	c.350_351del	Ala117Glyfs*43	PT	Ex 2	c.307_308del	Arg103Alafs*16	PT	/									[25, 34]
Ex 2	c.365G > T	Arg122Leu	MS	Ex 2	c.322G > T	Asp108Tyr	MS	/									[82]
Ex 2	c.377C > G	Pro126Arg	MS	Ex 2	c.334C > G	Arg112Gly	MS	/									[25]
Ex 2	c.378_380dup	Ser127dup	In-frame INS	Ex 2	c.335_337dup	Arg112dup ^f^	In-frame INS	/	/			/				non-Hodgkin’s lymphoma, cervical ca., phyllodes tumor	[10, 25]
Ex 2	c.382_383delinsCT	Ala128Leu	MS	Ex 2	c.339_340delinsCT	Pro114Ser	MS	/									[63]
Ex 2	c.383_398del	Ala128Glufs*39	PT	Ex 2	c.340_355del	Pro114Argfs*27	PT		/								[83]
Ex 2	c.*2del	–	3’UTR	Ex 2	c.358del	Glu120Serfs*26	PT	/				/		/			[25, 67]
Ex 2	c.*21 T > A	–	3’UTR	Ex 2	c.377 T > A	Val126Asp	MS	/	/								[4, 9, 32, 48, 69, 76]
Ex 2	c.*23G > C	–	3’UTR	Ex 2	c.379G > C	Ala127Pro	MS	/	/								[52, 55, 84, 85]
Ex 2	c.*101G > T	–	3’UTR	Ex 2	c.457G > T	Asp153Tyr	MS	/	/								[32]
In 2	c.*102-105A > G	n.d.	3’UTR	In 2	c.458-105A > G	n.d.	Int	/									[25]
	Del part of Exon 2	n.d.	PT		Del of Exon 1 & part of Exon 2			/									[86]
	Del of entire CDS	–			Del of entire CDS			/		/			/		/	GBM, astrocytoma, meningioma	[12, 16]

*Ex* exon, *In* intron, *CDS* coding sequence, *Del* deletion, *PT* protein truncating, *MS* missense, *INS* in-frame insertion, *Int* intronic, *3UTR* 3-prime UTR, ca. cancer, *Mel* melanoma, *PCa* pancreatic cancer, *NST* neural system tumors, *Sarc* sarcoma, *BrCa* breast cancer, *HNSCC* head and neck squamous cell carcinoma, *LCa* Lung cancer, *NFB* neurofibroma, *Uvl* uveal melanoma, *NHL* non-Hodgkin’s lymphoma, *GBM* glioblastoma multiforme, *n.d.* not determined

^a^ Splice site alteration is experimentally shown to result in protein truncation through exon skipping

^b^ Alternative names for NM_000077:c.-16_8dup (p.Ala4_Pro11dup) in the literature: 1_24dup, 23ins24, 32ins24, c.9_32dup24, c.24_47dup24, c.32_33ins24, c.32_33ins9_32, 24 bp duplication/insertion, p.M1_S8dup, p.1_8dup8

^c^ Alternative name for NM_000077: c.148C > T (p.Gln50*) in the literature: 50Q > X

^d^ Alternative name for NM_000077: c.172C > T (p.Arg58*) in the literature: p16INK4a Arg50Ter

^e^ Alternative name for NM_000077: c.225_243del (p.Ala76Cysfs*64) in the literature: p16-Leiden

^f^ Alternative names for NM_000077: c.335_337dup (p. Arg112dup) in the literature: 337-338insGTC, 113insR, 113insArg, p.R112_L113insR, 112-113insArg, p.Arg105ins

Based on the distribution of reported neoplasms with germline *CDKN2A* variants in Table 1, the association with melanoma is evidently irrespective of variant consequence on both p16^INK4A^ and p14^ARF^. Variants affecting p16^INK4A^ coding transcript are more frequently observed with pancreatic cancer and HNSCC (23/55) compared to p14^ARF^ (8/29). This association is supported by an analysis of Dutch melanoma families demonstrating pancreatic cancer events in 58% of families with p16^INK4A^-affecting variants but none among p14^ARF^-affecting carrier families [87]. Intriguingly, a broader spectrum of cancers – e.g. uterine cancer, NSTs, GBM, non-Hodgkin’s lymphoma – is noted to co-occur with p14^ARF^-affecting variants. Moreover, variants with a loss-of-function consequence exclusive to p14^ARF^, namely deletion of exon 1β, Glu33Glyfs*30 and Arg88*, were observed in individuals with adenocarcinomas of uterus, bladder and stomach, respectively. This apparent distinction of cancers observed with predicted loss either of p16^INK4A^ or p14^ARF^ function is congruent with the independent roles of both tumor suppressors in regulation of cell cycle progression and p53 pathway. In particular, the range of neoplasms co-occuring with p14^ARF^ variants is reminiscent of Li-Fraumeni syndrome, which is characterized by constitutional mutations in *TP53* and diminished p53 activity. Indeed, a dysregulated p53 pathway was observed exclusively in the malignant peripheral nerve sheath tumor (MPNST) of a germline *CDKN2A* deletion carrier diagnosed with synchronous HNSCC and MPNST [16]. It is also noteworthy that manifestations of neural system-related tumors such as MPNST, GBM, astrocytoma, and schwannoma were consistently reported together with families harbouring gross deletion of the *CDKN2A* locus and/or involving loss of an intact p14^ARF^ [12, 13, 16, 62, 88], suggesting a constitutional deficiency of p14^ARF^ associated with NSTs.

Collectively, these observed trends imply that *CDKN2A*-associated cancer susceptibility could be dependent on molecular consequence of the variant and affected transcript. While inferring this genotype-phenotype relationship is currently limited by the potential bias resulting from a p16^INK4A^-centric focus in *CDKN2A*-related literature, an appreciation for this distinction in p16^INK4A^/p14^ARF^ and larger case-cohort studies designed to address the causal effect of the specific variants will provide clarity in the future.

### Implications on clinical management

Presently, clinical genetic testing for *CDKN2A* is indicated for individuals with multiple primary melanoma and/or a family history of melanoma or pancreatic cancer [89]. However, the expanded spectrum of phenotype accompanying germline alterations in *CDKN2A* suggests that it may be relevant to consider *CDKN2A* as a candidate for tumor predisposition beyond melanoma and pancreatic cancer in clinical practice. Indeed, numerous carriers of pathogenic/likely pathogenic variants (P/LPV) listed in Table 1 reported a variable family history of cancers, including sarcoma, leukemia, lymphoma, astrocytoma and cancers of the breast, lung, and prostate. It has been alluded that constitutional deficiency in *CDKN2A* phenotypically mirrors the broad tumor spectrum characteristic of Li-Fraumeni syndrome [13, 16, 18, 90], hence clinicians and genetic professionals should consider *CDKN2A* as a differential diagnosis for cancers such as HNSCC, NSTs, breast cancer, and sarcomas. One potential approach is to evaluate at-risk individuals with an assessment tool built upon a scoring system that accounts for the spectrum of personal and family history of cancers, such as one proposed tailored-approach for clinical management of hereditary melanoma [91]. Additionally, it is important to be mindful that identification of *CDKN2A* genetic alterations has been historically restricted to the p16^INK4A^ transcript, which would exclude the alternative coding region specific to p14^ARF^ (i.e. exon 1β). This could result in missed diagnoses especially for neoplasms potentially driven by p14^ARF^ deficiency, therefore it is imperative that genetic professionals comprehensively interrogate for alterations in both transcripts.

Current guidelines for clinicians managing individuals tested positive for *CDKN2A* germline P/LPV are directed towards surveillance for melanoma and pancreatic cancer. Carriers are recommended to undergo bi-annual comprehensive skin examination including scalp and genitalia by a dermatologist, supplemented with total body photography and dermoscopy [92, 93]. Earlier detection of melanoma and non-melanoma skin cancers have been demonstrated among carriers compliant to surveillance [94, 95], although larger cohort studies will be required to better evaluate the outcomes and factors influencing successful melanoma screening. Annual pancreatic surveillance with contrast-enhanced magnetic resonance imaging and/or endoscopic ultrasound is recommended for *CDKN2A* pathogenic variant carriers beginning age 40 years regardless of family history given their high lifetime risk [96] and emerging evidence supporting the potential for downstaging and improved 5-year overall survival [97–99]. Patients are also encouraged to adopt lifestyle modifications to reduce cancer risk, including regular exercise, healthy diet, limiting alcohol intake, practicing sun-smart behaviour and smoking cessation. Healthcare professionals caring for *CDKN2A* carriers should have a heightened index of suspicion for malignancies beyond melanoma and pancreatic cancer. Although there are currently no formal recommendations for surveillance beyond melanoma and pancreatic cancer, clinicians should monitor the presentation of neoplasms within patients’ families and consider individualized discussion on the risk and benefit of screening, especially for prevalent cancers. Additionally, at-risk family members should be offered familial genetic testing given that up to 44% of relatives of index patients carry the familial variant, of whom 96% were observed to comply with surveillance [100]. Considering the broad range of management strategies, a multidisciplinary approach to care through a centralized cancer genetics service will benefit these patients [101].

With the rapid uptake of multigene panel testing in clinical setting, new data will continuously re-frame our understanding on the genotype-phenotype associations relevant to *CDKN2A*. This is exemplified by a recent analysis evaluating the clinical phenotype and molecular results of hereditary cancer predisposition testing in 165,000 individuals, which revealed an association of germline *CDKN2A* pathogenic variants with increased risk for breast cancer (odds ratio: 3.35, 95% CI: 1.43–7.75) [102]. Clinicians should keep abreast with the constant updates given that this is an evolving field and that clinical management of individuals harbouring germline *CDKN2A* variants will likely recalibrate with time.

## Conclusion

Cancer susceptibility among germline variant carriers of *CDKN2A* extend beyond the well-known predisposition to melanoma and pancreatic cancer, potentially associated with a multitude of cancers. The spectrum of associated cancer types may be driven by specific molecular consequences on p16^INK4A^ and/or p14^ARF^, warranting validation in future studies. Clinicians and genetic professionals should be cognizant of this expanded range of phenotypes and consider *CDKN2A* as a candidate gene for tumor predisposition syndrome in individuals and families presenting with such broad spectrum of cancers.

## Data Availability

No new data or material. The data used and analysed in this review are derived from ClinVar (https://www.ncbi.nlm.nih.gov/clinvar/) and cited publications.

## Abbreviations

bp: Base pairs
CI: Confidence interval
GBM: Glioblastoma multiforme
HNSCC: Head and neck squamous cell carcinoma
kb: Kilobase
MPNST: Malignant peripheral nerve sheath tumor
NST: Neural system tumor
P/LPV: Pathogenic/likely pathogenic variants
